# The Use of Iodine as a Stomachic Sedative

**Published:** 1883-06-20

**Authors:** Thomas T. Gaunt

**Affiliations:** New York


### THE USE OF IODINE AS A STOMACHIC SEDATIVE.

BY THOMAS T. GAUNT, M. D., OF NEW YORK.

The employment of iodine for the relief of the vomiting of pregnancy has been somewhat in vogue for a number of years. And while the success attending its use has been pointed out with more or less enthusiasm by a number of observers, its exact value has never been established, and so far as I have been able to ascertain, it is more often employed in actual practice, as a *dernier resort*, than as a remedy of much promise.

Some three years ago I began using small doses of the compound tincture of iodine as a stomachic sedative, and have gotten such satisfaction from its employment, that I have thought the appended cases might prove of possible interest to those in the medical profession who seek for more reliable means of controlling persistent vomiting than the routine measures usually employed for the relief of this most distressing symptom. So far as I have been able to discover from a careful review of most of the standard works, iodine has not been put upon record as of value for relieving other forms of vomiting than that of the pregnant state.

The first occasion on which I used iodine as a stomachic sedative was in the following case:

Case I.—A woman, aged 50 years, had been suffering for two hours from severe vomiting which accompanied an acute indigestion. I ordered her three drops of the compound tincture of iodine, in a tablespoonful of water, every fifteen minutes until relieved. After taking the second dose the vomiting ceased, and before fifteen minutes more she was sleeping soundly. The following morning she awoke without the slightest feeling of nausea and was able to eat a hearty breakfast with relish.

I was so favorably impressed with the action of the remedy in this case that I decided to give it a further trial. The next suitable case which presented an opportunity for investigating its value, was the following:

Case II.—A woman, aged 43 years, had been suffering for a number of weeks from irregular rigors followed by a variable rise of temperature and profuse sweating. Annoying as these symptoms were, she considered them trifling when compared with the constant nausea and frequent vomiting from which she also suffered, and for the relief of which I saw her in consultation. We were, at the time, unable to make a diagnosis as to her general condition; but later, from the subsequent history, concluded that she was suffering from acute general miliary tuberculosis. Faithful efforts had been made to control the vomiting, both by a variety of drugs, and by so arranging her diet as might best conserve for the relief of this distressing symptom, which was depriving the patient of both food and sleep. I advised that she should be given five drops of the compound tincture of iodine every four hours. This was done, and in less than thirty-six hours she was relieved of both nausea and vomiting.

Following up these cases, in which iodine had given good results, I began to give this remedy in a large number of cases of vomiting from a great variety of causes. For example: I have used iodine in doses of from one to five drops, frequently administered, for the vomiting of phthisis. During the past year I have employed it over fifty times, and find it to give better results than any other remedy that I have seen used for this purpose. The following case is a fair example of the satisfaction which may be had from the use of small doses of iodine for relieving phthisical vomiting.

Case III.—A woman, aged 28 years, who had catarrhal phthisis which had advanced to the stage of excavation, had for three weeks before I saw her, been suffering from almost constant vomiting, having been unable to retain even the simplest nourishment during this time, in consequence of which she had grown very anæmic, emaciated rapidly, and her other general symptoms had become much aggravated. I ordered her to have two drops of the compound tincture of iodine every fifteen minutes until relieved. In less than two hours after taking the first dose the vomiting had ceased, and when I saw her again, at the end of one week, she was eating fairly well, was able to be about with comfort, and was withal better than she had been for a number of weeks previously.

This rapid checking of emesis, in a class of cases usually so difficult to control, is a fair example of the power possessed by iodine in checking vomiting, and it is easy to infer therefrom the comfort afforded patients, and the satisfaction one gets from the use of this drug in the vomiting of phthisis. In fact I have found iodine, in small doses, to exercise such a particularly salutary influence over phthisical vomiting, that I wish to record my belief that we have in it the most generally useful agent at our command for controlling this very distressing and obstinate symptom.

I have been able to check the annoying vomiting sometimes seen in the following case :

CASE IV.—A woman, aged 35 years, who had been subject occasionally to attacks of hysteria about the menstrual epoch. Two months previous to the present attack she had vomited for three weeks, commencing with the menstrual flow, and ending two weeks before the next period. During this time she had been confined to bed, and given various remedies without relief. She finally recovered after all medication had been discontinued. Six weeks after this, she again began to menstruate, and shortly after to vomit profusely. I gave her two drops of the compound tincture of iodine every half hour, from which she said she experienced great relief, and in less than forty-eight hours she ceased vomiting completely.

We all know that conclusions based on the use of drugs in hysterical women are, as a rule, fallacious, and I only cite this as one out of several cases of hysterical vomiting in which iodine has afforded me much satisfaction.

The morning nausea and vomiting of drunkards, as well as the more persistent emesis, from which they often suffer after a prolonged debauch, have, in my experience, yielded more readily to small doses of iodine, than to any of the somewhat lengthy list from which selection is usually made.

CASE V.—A man, aged 45 years, who had been a hard drinker all his life, always after a prolonged indulgence suffered from annoying vomiting for several days. He consulted me while suffering from an attack of unusual severity. By giving him three drops of the compound tincture of iodine every hour, I was able to quickly stop his vomiting, and at the end of twenty-four hours he was feeling so well that he began again to drink as freely as before.

The foregoing is the last one of a long series of similar cases, and I cite it not as a striking illustration, but, because it has come under my care while writing this.

The vomiting we so frequently meet with in severe cases of septicæmia is very harassing to the patient, and renders the prognosis so much more grave, that it behooves us to check it as quickly as possible. I have had several cases of septicæmia under my charge during the past few months, and have been greatly pleased with the control which small, frequently repeated doses of iodine seemed to exert over the symptom.

The results in the following case were particularly gratifying.

CASE VI.—A woman, aged 30 years; I was asked to take charge of this patient immediately after she had been operated on, and had her under my care for two weeks. The patient was suffering from phthisis, and had been jaundiced for six months before the operation was done. The operation consisted in a resection of the right ankle. The details of the case have no interest for us here; suffice it to say, that, notwithstanding careful antiseptic treatment of the wound, the woman developed marked signs of septic poisoning, and before the end of a week had a temperature of 105°, and was rejecting everything swallowed. After trying to establish a tolerance of the stomach by a regulated diet and failing, I gave her one drop of the compound tincture of iodine every fifteen minutes. In less than six hours I was able to control the vomiting, and by means of an occasional dose of iodine kept the irritability of the stomach perfectly in check. At the end of the following week I delivered up the patient who was eating and sleeping well, with a temperature but slightly elevated and the wound doing finely.

I feel confident, that had it not been for the iodine which checked the vomiting, the patient would have been shortly reduced to a condition of extreme prostration. For, judging by a large number of cases of septicæmia which I have seen, we can have but slight hope of checking the vomiting from the use of the routine anti-emetic remedies.

In the latter stages of chronic nephritis, where we so often encounter troublesome attacks of vomiting, I have used small doses of iodine with good success. The benefit obtained is often lasting, but I now and again come across a case where the administration of iodine has to be kept up for a considerable time, and later used whenever nausea and vomiting again supervene.

CASE VII.—A man, aged 55 years, has chronic diffuse nephritis, and is suffering from almost constant nausea and vomiting. Gave him two drops of the compound tincture of iodine every four hours. This checked the vomiting and relieved the nausea. By taking an occasional dose of iodine whenever vomiting threatened he was able to keep the irritability of his stomach in abeyance.

It would appear at first sight a doubtful expedient to give iodine in even comparatively small doses to patients with acute catarrhal gastritis. I have often given it in this condition, however, with the best satisfaction, as the following case may serve to illustrate.

CASE VIII.—A man, aged 50 years, suffering from acute catarrhal gastritis. When I saw him he was having free, but somewhat painful emesis. I gave him one drop of the compound tincture of iodine every fifteen minutes, and had the pleasure of seeing the vomiting stop after he had taken three or four doses of the medicine.

But I do not intend to give examples of all the classes of cases in which I have used iodine as a stomachic sedative, for did I attempt this, I should be obliged to far exceed the limits I have proposed for this paper.

I will conclude with some half-dozen cases, chosen at random from a series of over one hundred cases of gastro-intestinal disturbance accompanied with vomiting which I have treated at the New York Dispensary, during the past summer, with drop doses of the compound tincture of iodine. The first case I will mention is one of gastritis with the following history.

CASE IX.—A child, aged 5 years, had been suffering with great pain in epigastrium and constant vomiting, with marked increase of temperature, for forty-eight hours before I saw him. The pain and vomiting had deprived the patient of rest, and together with the increased temperature and impossibility of retaining food, or even water, had caused him to emaciate rapidly. He was greatly prostrated when I was first called to see him. I ordered him one drop of the compound tincture of iodine every fifteen minutes, and instructed the mother to allow him a few drops of a mixture of equal parts of milk and lime-water, as soon as his stomach showed a tolerance of the iodine. At the end of two hours the medicine was retained, as was also the milk, which was given in small but increasing quantities, and at frequent intervals. This child went on to rapid convalescence, and had no return of the vomiting after it was at first checked by the iodine.

I prefer the following form for administration—

| | | |
|---|---|---|
| ℞ | Tincturæ iodinii compositæ | ♏ viij–ʒ ss, |
| | Bismuthi subnitratis | ʒ j–℈ j, |
| | Glycerinæ, | aa ʒ iij, |
| | Aquæ cinnamomi | |
| | Liquoris calcis, ad | ℥ ij. |

M. et Sig. Dose, one dessertspoonful.

The mixture is to be well shaken up before being taken. Give a dessertspoonful every fifteen minutes until the vomiting ceases.

It may be objected that the bismuth and lime-water are the active ingredients in this formula. That this is not true, any one may prove to his own satisfaction, by studying the effect of the compound tincture of iodine, when given in drop doses dissolved in pure water. I find, however, that by using the above combination, the bismuth and lime-water help to allay the irritability of the stomach, and when they are given in conjunction with iodine, I think that the effect of the latter are more lasting, while the nausea is certainly controlled more quickly. Should intestinal disturbance also exist, the bismuth of course is still further advantageous. This prescription needs to be prepared anew every day, as the free iodine combines with the lime to form the iodide of calcium, on standing twelve hours.

Or, what will be found more convenient, have the above formula put up but omit the iodine, which is to be kept in a separate bottle, and the requisite amount dropped in the dessertspoonful immediately before administration.—*Ame Jour. Med. Science.*

